# Supplementary material for: Effects of intermittent hypoxia on adipose-derived mesenchymal stem cells in protecting alveolar type II cells from injury
Source: PLoS One. 2026 Jan 21;21(1):e0340291. doi: 10.1371/journal.pone.0340291 (PMC12822967; doi:10.1371/journal.pone.0340291)
Supplement: S1 File — The raw data from Western blot. (PDF) [file pone.0340291.s001.pdf]

Bcl-2, relative gray value:

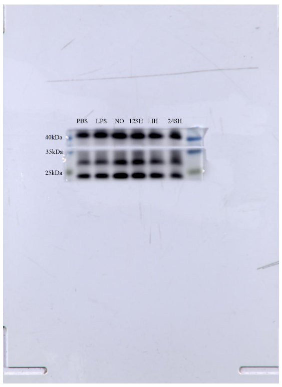

0.919662998
0.801953115
0.910344243
0.925337292
0.94245429
0.915035452

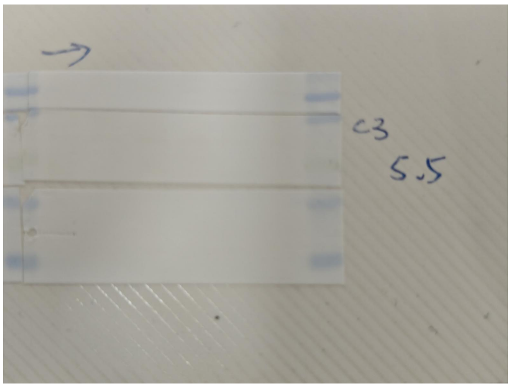

Bax, relative gray value:

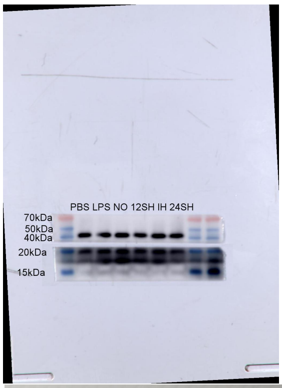

1.0113058
1.244036751
0.994243348
1.011036601
0.961209301
0.832362052

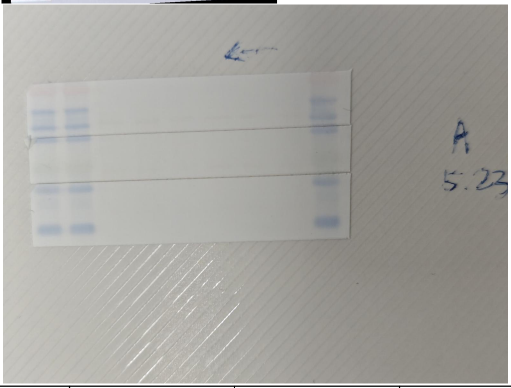

**Bax/Bcl-2**	1.099648243	1.551258705	1.092161955	1.092614131	1.019900181	0.909650058

Bcl-2, relative gray value:

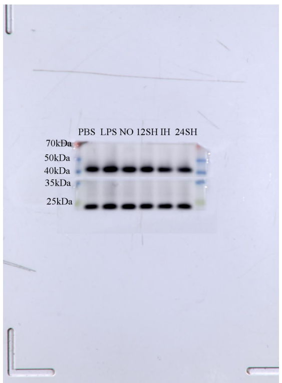

0.95363099
0.873443835
0.997139731
0.943676563
1.124930435
1.168779654

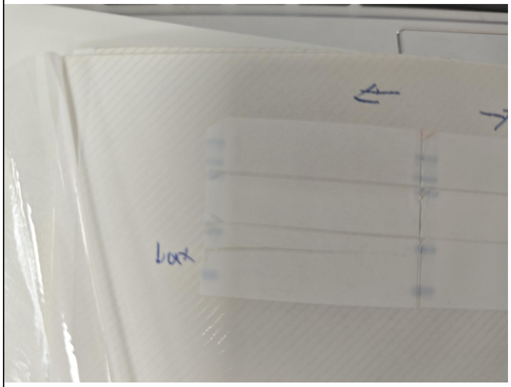

Bax, relative gray value:

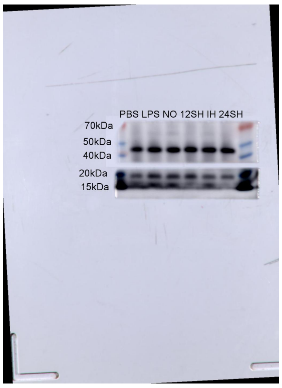

0.940172235
1.084257268
1.05791081
0.954594646
0.662656256
0.603648705

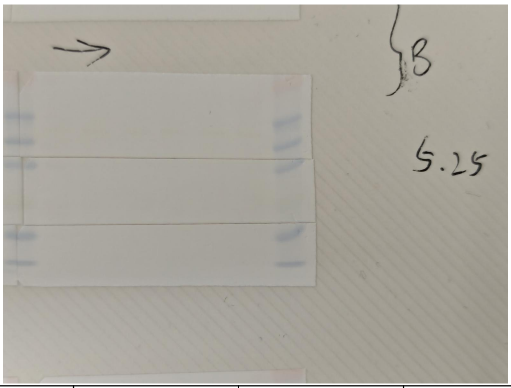

**Bax/Bcl-2**	0.985886831	1.241358888	1.060945399	1.01156973	0.589064208	0.516477766

Bcl-2, relative gray value:

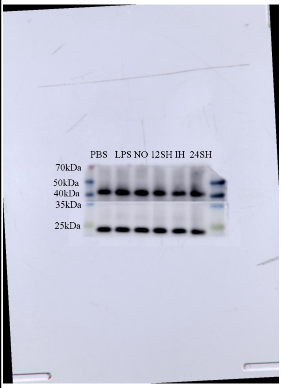

1.078085338
0.896160865
0.926956857
1.054873634
1.137584954
0.934020161

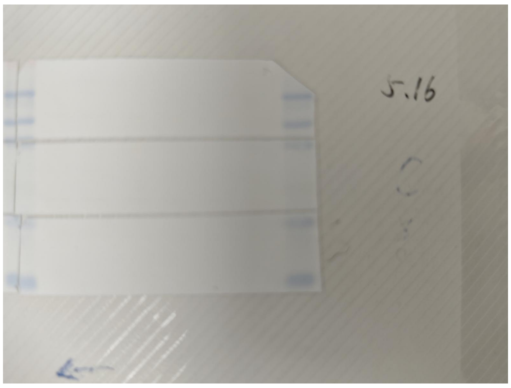

Bax, relative gray value:

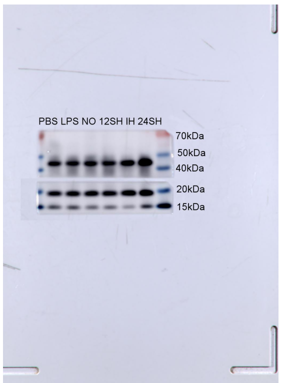

1.000094652
1.038597194
1.082686662
1.061322514
1.088249385
0.941041101

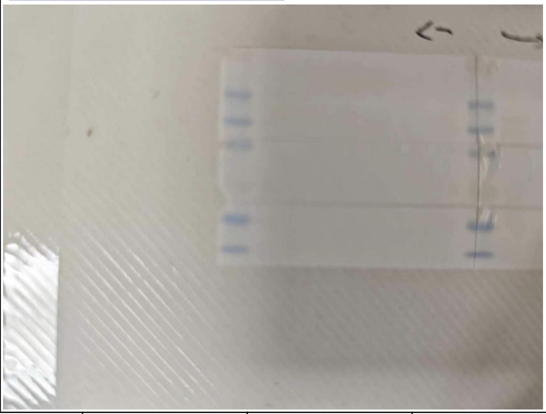

**Bax/Bcl-2**	0.927658152	1.158940581	1.168001136	1.006113415	0.95663131	1.007516904

Bcl-2, relative gray value:

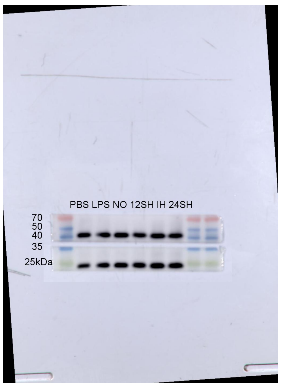

0.732603764
0.879106439
0.853206699
1.007944715
0.984109255
0.981939277

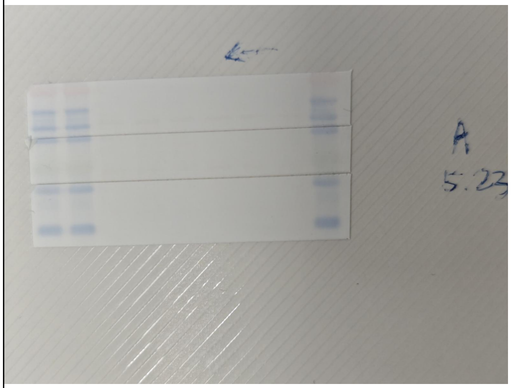

Bax, relative gray value:

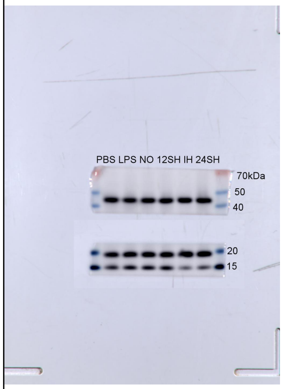

0.824757846
1.018842034
0.915933572
0.903641762
1.063409992
0.967668705

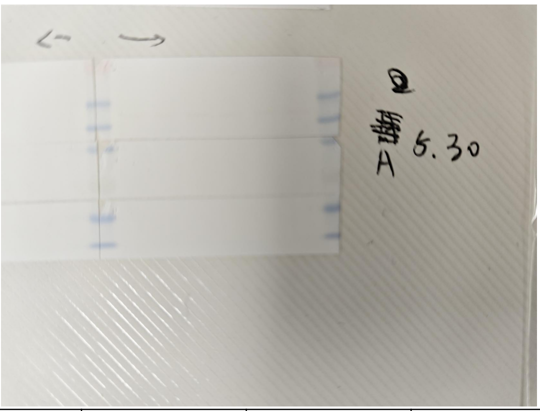

**Bax/Bcl-2**	1.125789801	1.15895185	1.073518964	0.896519173	1.080581233	0.98546695

Bcl-2, relative gray value:

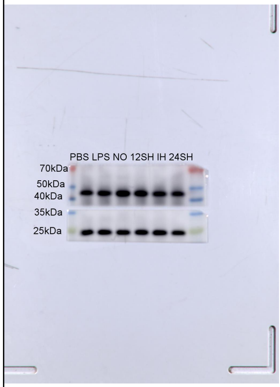

1.12353464
1.068202873
0.871650441
0.942508159
1.097533224
1.070622738

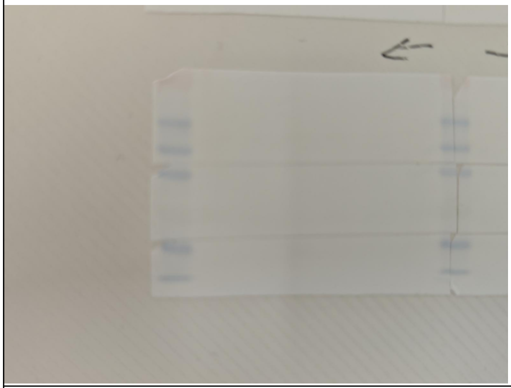

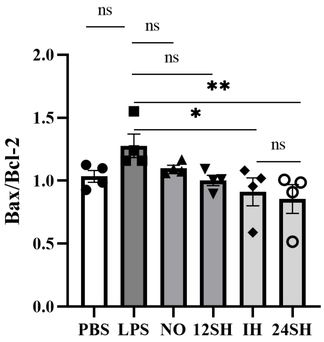

LPS vs. PBS	ns	0.1612
LPS vs. NO	ns	0.3994
LPS vs. 12SH	ns	0.0943
LPS vs. IH	*	0.0188
LPS vs. 24SH	**	0.0064

HIF-1α, relative gray value(Protein loading amount: 30 μg):

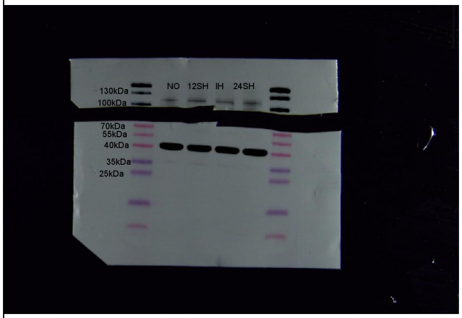

0.475387493
0.692917098
0.672477107
0.698905764

HIF-1α, relative gray value(Protein loading amount: 40 μg):

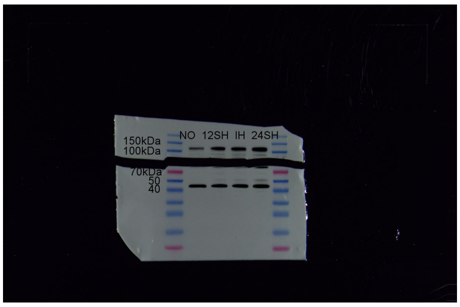

0.307392342
0.769679622
0.8112282
0.94462018

HIF-1α (Protein loading amount: 40 μg):

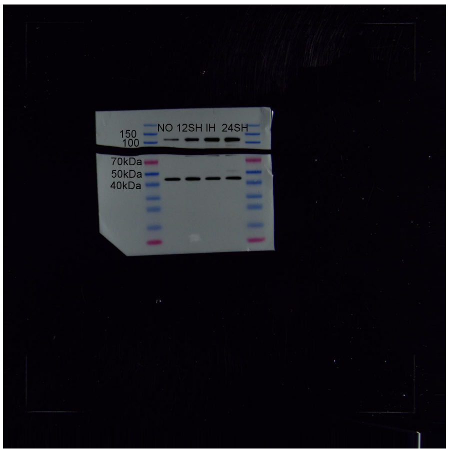

0.586924715
0.840796396
1.094741404
1.110028145

HIF-1α (Protein loading amount: 20 μg):

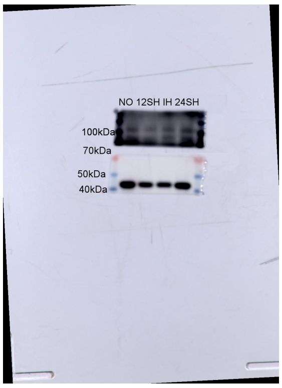

0.454005993
0.550114686
1.14158049
0.913629528

HIF-1α, relative gray value (Protein loading amount: 20 μg):

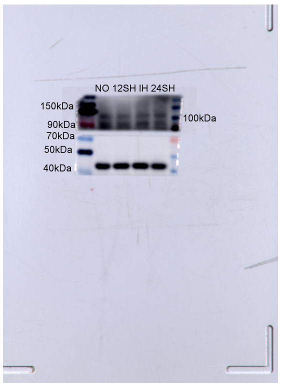

0.791462007
0.817225237
0.714194296
0.882538287

Statistical chart

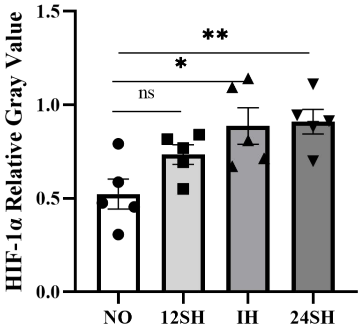

NO vs. 12SH	ns	0.1589
NO vs. IH	*	0.0100
NO vs. 24SH	**	0.0064

Cleaved Caspase-3/Caspase-3 :

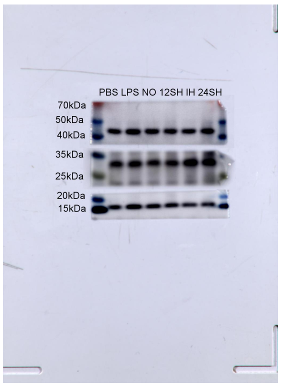

0.922503604
1.334004152
1.263621487
1.04538816
0.653870553
0.75246314

Cleaved Caspase-3/Caspase-3 :

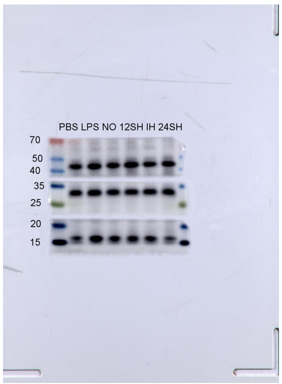

0.685590263
0.946265894
0.863332809
0.844500171
0.802864284
0.615493236

Cleaved Caspase-3/Caspase-3 :

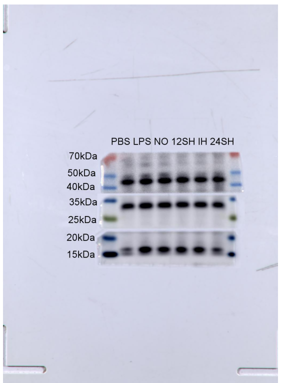

0.498950716
0.943116653
0.842475244
0.855320951
0.856051628
0.546172361

Cleaved Caspase-3/Caspase-3 :

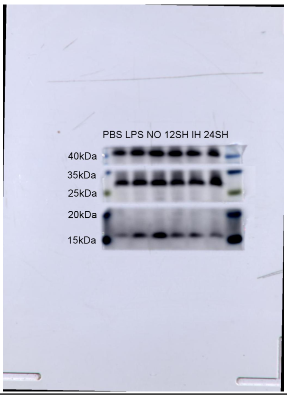

0.431645776
0.875291044
1.05522558
0.724005118
0.616639376
0.423276085

CleavedCaspase3/Caspase3 :

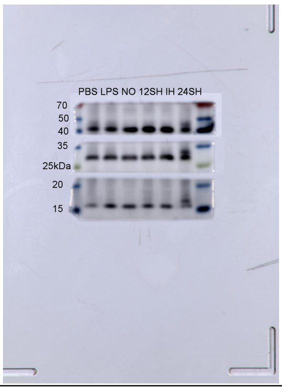

0.59442798
1.031052154
0.970708895
0.843178194
0.746287773
0.65458608

CleavedCaspase-3/Caspase-3 :

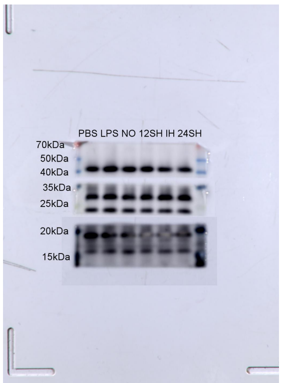

0.712684893
0.802294906
1.074739246
0.843543695
0.747092374
0.77366422

CleavedCaspase3/Caspase3 :

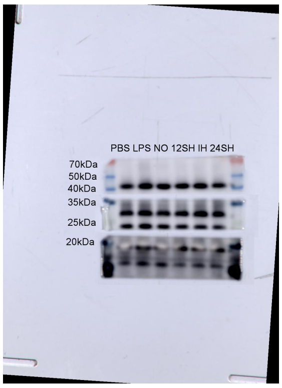

0.801211185
0.832608815
0.963869805
0.611543753
0.717378816
0.730028683

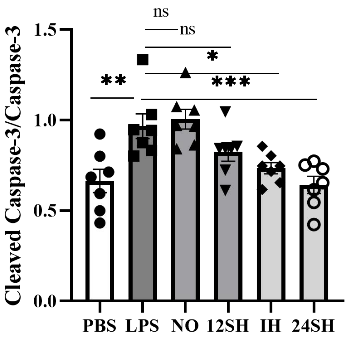

LPS vs. PBS	**	0.0015
LPS vs. NO	ns	0.9816
LPS vs. 12SH	ns	0.2402
LPS vs. IH	*	0.0186
LPS vs. 24SH	***	0.0007

Cleaved Caspase-3:

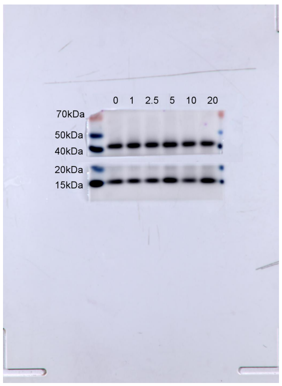

0.842969858
0.716751276
0.788986372
0.960679514
0.720239285
1.124954616

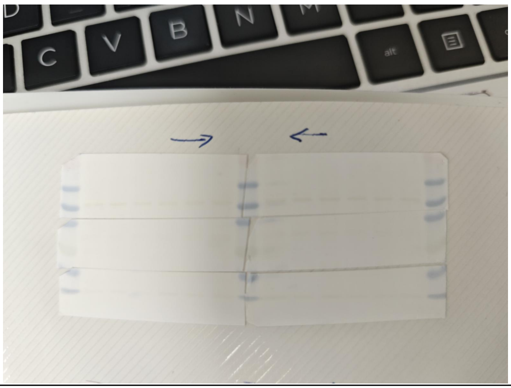

Cleaved Caspase-3:

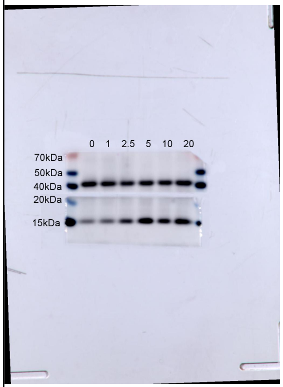

0.268944605
0.371903041
0.740814766
1.121091643
0.804104991
0.900130025

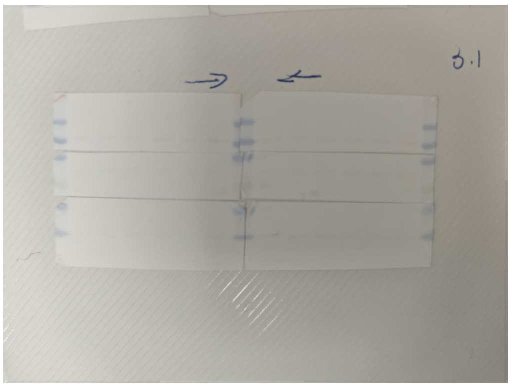

Cleaved Caspase-3:

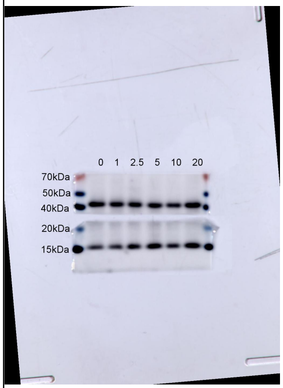

0.60180441
0.672611361
0.936918791
1.158064701
0.865820187
0.917063728

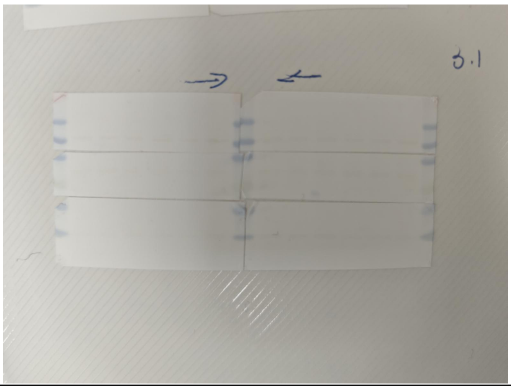

Cleaved Caspase-3:

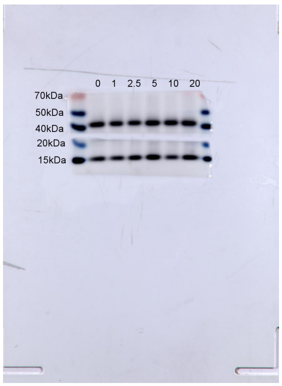

0.753140054
0.82097018
1.044685415
0.964879238
0.651761919
0.943170108

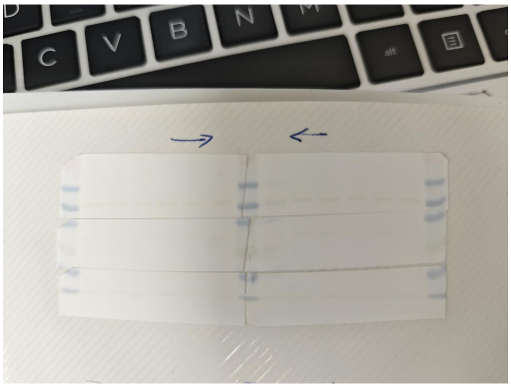

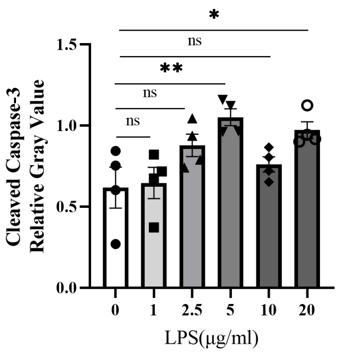	No total caspase-3 results due to unresolved overexposure issue at the time.<br>0 vs. 1 ns 0.9986<br>0 vs. 2.5 ns 0.1148<br>0 vs. 5 ** 0.0047<br>0 vs. 10 ns 0.5854<br>0 vs. 20 * 0.0217
