# Supplementary material for: Effects of intermittent hypoxia on adipose-derived mesenchymal stem cells in protecting alveolar type II cells from injury
Source: PLoS One. 2026 Jan 21;21(1):e0340291. doi: 10.1371/journal.pone.0340291 (PMC12822967; doi:10.1371/journal.pone.0340291)
Supplement: Attachment — Submitted filename: Response to Reviewers.docx [file pone.0340291.s004.docx]

Dear Reviewers:

We sincerely appreciate your recognition, comments, and suggestions concerning our study. We identified an error in the sequential entry of grayscale values for the cleaved caspase-3/caspase-3 data and Bax/Bcl-2 from different sample dates. This error has now been corrected in the figures. We have removed the sections of the JC-1 statistical graph where the data volumes differed among groups. We have redone the figures to fully display the P-values for different groups and added new images from supplementary experiments (for the identification of adipose-derived stem cells, ADSCs) (although it may not be apparent in the Word document's revision mode, the modified parts of the figures have been attached at the end of this paper). And we have carefully considered each point for your comments, and hereby present our detailed replies as follows (The line numbers we refer to are all from the "Revised Manuscript with Track Changes" document):

**To Reviewer 1:**

1. **The hypoxia protocols are not fully specified. For full reproducibility, please state ramp time to each O₂ setpoint. A brief note on how O₂ was monitored would be better.**

 our incubator automatically regulates the oxygen concentration within the culture system based on both the preset oxygen level and the oxygen concentration in the gas cylinder, achieving the target oxygen concentration within 1 minute. The revisions are made in Section 2.2 of the manuscript, specifically on lines 104-106.

Our incubator is equipped with an oxygen concentration monitoring probe inside. It can directly display the oxygen concentration on the electronic screen outside the incubator. Moreover, the oxygen concentrations of the customized gas cylinders we've ordered are 5% and 21% respectively. The oxygen concentrations for modeling and at each time point can be directly viewed on the electronic screen of the incubator. In fact, within the first 30 minutes of hypoxic conditions, the oxygen concentration inside the incubator fluctuates between 4.5% and 5.5%, but the overall baseline remains essentially at 5%. During the 30-minute period of normoxic conditions, the oxygen concentration inside the incubator gradually decreases from an initial 21% to 18%, the value of 20% mentioned in our manuscript represents the average.

2. **IH also shows lower VEGF and stronger GSH recovery. However, causal tests are not included. Emphasize correlation rather than superiority and add an appropriate discussion of this point in the Discussion section.**

Thank you for pointing out my mistakes. We have revised the wording in the manuscript, specifically on lines 318-324: the decrease in VEGF in IH - ADSCs - CM did not affect its therapeutic efficacy, indicating that the specific anti - apoptotic mechanism of ADSCs - CM may have a weak association with VEGF. We believe that this decrease in VEGF may not necessarily be a negative thing, but it still requires further in vivo studies for verification. in the discussion section.

And on lines 352-355: in this study, the decrease in VEGF in IH - ADSCs - CM did not affect its therapeutic efficacy, indicating that the specific anti - apoptotic mechanism of ADSCs - CM may have a weak association with VEGF. We believe that this decrease in VEGF may not necessarily be a negative thing, but it still requires further in vivo studies for verification.

3. **In Results 3.4, flow cytometry indicates reduced apoptosis across all CM groups, while western blots show no significant improvement with NO or 12SH.**

We explain on lines 359-364 in the discussion section. We think it may be because flow cytometry has high sensitivity in detecting apoptotic cells and is capable of capturing early-stage apoptotic cells. The NO group and the 12SH group might have shown certain effects during the early apoptotic phase, but these effects did not persist to the time point of Western Blot detection. In flow cytometry, the excessively high proportion of early apoptotic cells obscured the overall trend.

4. **In multi-panel figures, the order is not fully consistent. Please revise either the figure or the text so that the first in-text callouts follow alphabetical order, and update the Figure legend to match the final sequence to optimize readability.**

We have revised the order of some images and their corresponding captions. Thank you for pointing out the issues.

**To Reviewer 2:**

1. **In the abstract, the authors should specify which cytokines were involved and indicate whether their levels increased or decreased.**

We have revised the abstract that “Finally, we concluded that Intermittent hypoxia preconditioning leads to decrease in the secretion of VEGF by ADSCs compared to sustained hypoxia preconditioning; but their therapeutic effects in terms of anti-apoptosis and anti-oxidation are comparable.”, as seen in lines 19-20 of the manuscript. Thank you for your reminder.

2. **In the introduction, the authors should introduce intermittent hypoxia preconditioning and discuss whether this condition has been studied using mesenchymal stem cells.**

We have revised the introduction section by adding research on intermittent hypoxia in mesenchymal stem cells at lines 59-66. In fact, there are various viewpoints regarding the research on intermittent hypoxia (IH) in stem cells. Some studies suggest that it can enhance osteogenic differentiation capacity^[1]^, while others claim it reduces it^[2]^. Some believe that IH can increase the secretion of cytokines such as hepatocyte growth factor (HGF) by stem cells^[3]^, whereas others argue that IH may impair the therapeutic potential of stem cells^[4]^. This discrepancy might be related to the different intermittent hypoxia treatment protocols employed. And our intermittent hypoxia model differs from those mentioned in the aforementioned studies.

3. **The authors should do some tests to make sure that the isolated ADSCs meet the minimum MSC criteria recommended by ISCT. First, they must adhere to plastic in standard culture conditions. Second, they must express a specific set of surface markers, namely CD105, CD73, and CD90, while lacking markers like CD45, CD34, CD14, CD11b, CD79a, CD19, and HLA-DR. Third, they must be able to differentiate into osteoblasts, adipocytes, and chondrocytes in vitro.**

We have supplemented the flow cytometry analysis of surface markers including CD34(0.35%), CD90(98.6%), and CD105(58.9%), Due to the replacement of the flow cytometry equipment midway, we selected the CD73, CD44, and CD45 results from another tube of cells that we had previously processed. Although there is a slight difference compared to the previous cell purity of around 97%, we can still consider the cell purity to be above 95%. Now, the remaining cells we have are only those from passages after the 7th generation. And we conducted adipogenic and osteogenic differentiation experiments on the cells. Using primary cells from the 7th passage, we were still able to successfully induce adipogenic differentiation on the 7th day of induction and osteogenic differentiation on the 18th day of induction. This should demonstrate that there are no issues with our cells' differentiation capabilities.

4. **The authors should also study the effects of intermittent hypoxia preconditioning on proliferation and stemness of ADSC.**

We actually attempted to investigate the proliferative activity of ADSCs under intermittent hypoxia (IH) conditions, but failed to obtain differential results in the CCK-8 cell viability assay, which we have added to Figure 2B in our manuscript. Additionally, we examined the cell cycle of ADSCs after modeling, but failed to obtain differential results, as shown in the following figure.

We believe that the results obtained so far confirm that there is no impact on proliferation. Ultimately, our focus shifted away from investigating the proliferative activity of ADSCs and towards exploring the therapeutic effects of their conditioned medium. Owing to the limitations imposed by time and cost, and, to a greater extent, the limited availability of primary cells, we are in a rather difficult position and have thus decided, with some reluctance, not to incorporate the exploration of how intermittent hypoxia preconditioning affects the stemness of ADSCs into the current experimental design. We kindly ask for your understanding.

5. **The authors should propose some next steps or future studies.**

Regarding the future research plan, we believe it is essential to continue investigating the impact of intermittent hypoxia on the proliferative and differentiative activities of ADSCs, as you suggested earlier (Add at lines 378 - 380). Moreover, we believe that future research should investigate the specific differences in the secretome of ADSCs induced by intermittent hypoxia. Additionally, the precise mechanism underlying the increase in GSH levels caused by their conditioned medium remains to be elucidated (lines 353-355). Furthermore, it remains uncertain why a decrease in VEGF was observed despite the fact that intermittent hypoxia indeed induced an elevation in HIF-1α. Although we have provided a degree of explanation regarding this, this outcome, which differs from previous studies, still awaits further exploration (lines 295-313).

Traces of image modifications

new

new

new

new

**Reference**

1.	Camacho-Cardenosa M, Pulido-Escribano V, Torrecillas-Baena B, Quesada-Gómez JM, Herrera-Martínez AD, Sola-Guirado RR, et al. Combined Effects of Cyclic Hypoxic and Mechanical Stimuli on Human Bone Marrow Mesenchymal Stem Cell Differentiation: A New Approach to the Treatment of Bone Loss. J Clin Med. 2024;13(19). Epub 20240928. doi: 10.3390/jcm13195805. PubMed PMID: 39407866; PubMed Central PMCID: PMCPMC11476683.

2.	Chen F, Gu M, Xu H, Zhou S, Shen Z, Li X, et al. Chronic intermittent hypoxia impairs BM-MSC osteogenesis and long bone growth through regulating histone lactylation. J Transl Med. 2025;23(1):845. Epub 20250728. doi: 10.1186/s12967-025-06849-w. PubMed PMID: 40722170; PubMed Central PMCID: PMCPMC12306074.

3.	Gharib SA, Dayyat EA, Khalyfa A, Kim J, Clair HB, Kucia M, et al. Intermittent hypoxia mobilizes bone marrow-derived very small embryonic-like stem cells and activates developmental transcriptional programs in mice. Sleep. 2010;33(11):1439-46. doi: 10.1093/sleep/33.11.1439. PubMed PMID: 21102985; PubMed Central PMCID: PMCPMC2954693.

4.	Ren J. Intermittent hypoxia BMSCs-derived exosomal miR-31-5p promotes lung adenocarcinoma development via WDR5-induced epithelial mesenchymal transition. Sleep Breath. 2023;27(4):1399-409. Epub 20221121. doi: 10.1007/s11325-022-02737-5. PubMed PMID: 36409397.
